# Vaccine Adjuvant Informatics: From Data Integration and Analysis to Rational Vaccine Adjuvant Design

**DOI:** 10.3389/fimmu.2014.00032

**Published:** 2014-02-03

**Authors:** Yongqun He

**Affiliations:** ^1^Unit for Laboratory Animal Medicine, University of Michigan Medical School, Ann Arbor, MI, USA; ^2^Department of Microbiology and Immunology, University of Michigan Medical School, Ann Arbor, MI, USA; ^3^Center for Computational Medicine and Bioinformatics, University of Michigan Medical School, Ann Arbor, MI, USA; ^4^Comprehensive Cancer Center, University of Michigan Medical School, Ann Arbor, MI, USA

**Keywords:** vaccine, adjuvant, bioinformatics, Vaxjo, systems vaccinology, vaccinomics, ontology, literature mining

A vaccine adjuvant is a vaccine component that is used to enhance host immune responses to the antigens in the vaccine. While live attenuated vaccines without adjuvants often stimulate strong immune responses, they are difficult to be approved for human use due to potential safety concerns. Subunit vaccines (e.g., protein and peptide vaccines) can purposely exclude possible allergens, toxins, and virulent molecular domains of a pathogen. The restriction of host immune responses to specified antigenic regions prevents autoimmune responses and increases vaccine safety. However, subunit vaccines do not typically induce strong immune responses. Therefore, the selection and usage of powerful vaccine adjuvants to enhance antigen-specific immune responses is critical to successful vaccine development.

**Figure 1:**
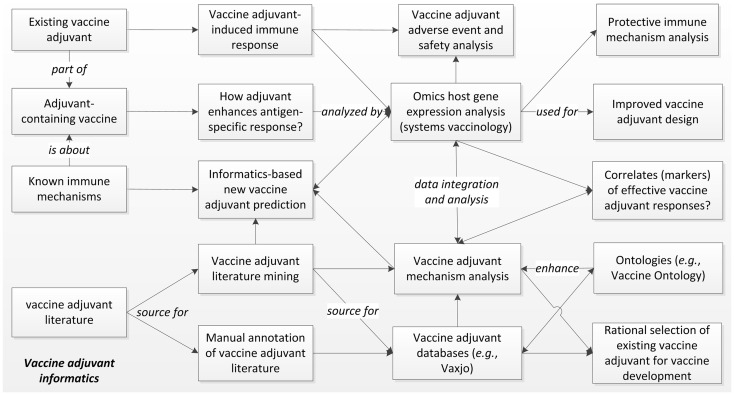
**The scope of vaccine adjuvant informatics research**. The immune responses and efficacy of existing vaccine adjuvants and adjuvant-containing vaccines can be specifically analyzed using high throughput Omics-based systems biology studies with the support of strong informatics data integration and analysis. These studies will elucidate the molecular mechanisms of protective or adverse immune pathways associated with vaccine adjuvants, identify gene markers of effective vaccine adjuvant responses, and support specific vaccine adjuvant and vaccine design. The large amount of vaccine adjuvant literature can also be utilized for manual and computer-assisted literature mining, leading to integrated mechanism analysis and database generation. The data can be logically represented and stored in biomedical ontologies that further improve literature mining. Vaccine adjuvant informatics also supports rational design of new adjuvants and selection of existing adjuvants for new vaccine development.

Intensive vaccine adjuvant research has been conducted and reported. As of January 11, 2014, a PubMed search of “vaccine and adjuvant” identified 35,000 articles published since 1948. Large amounts of high throughput gene expression datasets related to vaccine adjuvants are also publically available in centralized repositories, e.g., the NCBI Gene Expression Omnibus (GEO). These “big data” cannot be systematically analyzed without computational approaches. The specific field of developing and applying informatics tools for vaccine adjuvant research and development can be called “vaccine adjuvant informatics”. We have previously described the “vaccine informatics” that focuses on the development and application of bioinformatics methods for facilitating different aspects of preclinical vaccine research and development, clinical vaccine trials, and post-licensure vaccine safety and efficacy surveillance (1). As a vaccine component, vaccine adjuvants are usually studied together with vaccines. However, vaccine adjuvants are manufactured independently, and they can be studied individually for different purposes. For example, vaccine adjuvants are often administered to specific hosts *in vivo* or cells *in vitro* for analysis of their induced immune responses and adverse effects. Overall, vaccine adjuvant informatics addresses scientific questions different from those associated with the whole vaccines or other vaccine components (e.g., vaccine antigens). Figure 1 illustrates the major aspects of vaccine adjuvant informatics.

There exist various vaccine adjuvant informatics tools, including literature mining, database development, ontology data integration and analysis, and Omics bioinformatics data analysis. We have recently developed a web-based vaccine adjuvant database Vaxjo (http://www.violinet.org/vaxjo) (2). Vaxjo stores the information of over 100 vaccine adjuvants and approximately 400 vaccines that use the adjuvants. To improve data integration and automated reasoning, the vaccine adjuvant-related data have been logically represented in the Vaccine Ontology (VO) (3). Biological ontologies are sets of computer- and human-interpretable terms and relations that logically represent biological entities and how they relate to each other. VO has been found to be able to improve literature mining of gene–gene integration (3). Omics-based informatics is a crucial part of the “systems vaccinology” for systematic analysis of host immunity induced by vaccines (4) and vaccine components. Omics and informatics are also critical to the implementation of the “vaccinomics” strategy for personalized vaccine (including vaccine adjuvant) adverse event analysis and personalized vaccine development (5).

Informatics studies support basic and translational vaccine adjuvant research. Mechanistic understanding of how innate immunity is initiated and how it shapes adaptive B- and T-cell responses has significantly influenced the development of vaccine adjuvants. For example, as parts of innate immune system, the pattern-recognition receptors (PRRs), including Toll-like receptors (TLRs) and nucleotide-binding oligomerization domain (NOD) proteins, recognize the pathogen-associated molecular patterns (PAMPs) such as bacterial lipopolysaccharide. Many TLR agonists have been developed as vaccine adjuvants to bind to TLRs, promoting the maturation of antigen presenting cells (e.g., dendritic cells) and further induction of adaptive immune responses (6). However, TLR agonists may also cause severe adverse effects by stimulating the production and release of inflammatory cytokines. Omics-based systems biology experiments and bioinformatics data analyses are able to provide more insights into the mechanisms of the interactions between PRRs and PAMPs, their downstream pathways, and how innate and adaptive immune responses are specifically affected. Such studies require advanced data processing and statistical data analysis based on existing knowledge on gene functions and molecular interaction networks (1).

Many new research areas have not been well studied but deserve intensive exploration. For example, it is likely that the immune responses induced by vaccine adjuvants and antigens can be different but cooperative. The mechanistic differences and similarities between vaccine adjuvant- and antigen-induced host responses and how these two types of responses interact have not been carefully studied. The addressing of these questions using a combination of Omics, literature mining, and informatics methods will likely lead to the development of new and better vaccine adjuvants and vaccines. In addition, rational vaccine adjuvant design needs to consider the factor of what infectious microbe or disease a vaccine is targeted for. Our Vaxjo data analysis has identified many possible correlations between infectious microbial agents targeted by vaccines and the adjuvants used in the vaccines (2). Out of over 100 adjuvants, it is challenging to predict which adjuvants are optimal for developing disease-specific vaccines. Such a task can be solved by advanced computational and statistical algorithms.

In the exciting era of post-genomics and informatics, we also expect the advent of more advanced computational algorithms, easy-to-use software programs, and even theoretic breakthroughs that will provide researchers more capabilities to conduct vaccine adjuvant research and development.
